# Homograft durability after correction of pulmonary atresia and ventricular septal defect with or without systemic pulmonary collateral arteries

**DOI:** 10.1016/j.xjon.2021.09.025

**Published:** 2021-09-24

**Authors:** Pieter C. van de Woestijne, Jamie L.R. Romeo, Ingrid van Beynum, Maarten Witsenburg, M. Mostafa Mokhles, Ad J.J.C. Bogers

**Affiliations:** a Department of Cardiothoracic Surgery, Erasmus University Medical Centre, Rotterdam, The Netherlands; b Department of Pediatric Cardiology, Erasmus University Medical Centre, Rotterdam, The Netherlands; c Department of Cardiology, Erasmus University Medical Centre, Rotterdam, The Netherlands

**Keywords:** congenital heart disease, homograft, pulmonary atresia, right ventricular outflow tract reconstruction, BTS, Blalock–Taussig shunt, NPA, native pulmonary artery, PA, pulmonary atresia, PR, pulmonary regurgitation, PVR, pulmonary valve replacement, RVOT, right ventricular outflow tract, SPCA, systemic-to-pulmonary collateral artery, VSD, ventricular septal defect

## Abstract

**Background:**

Pulmonary atresia and ventricular septal defect (PA-VSD), with or without systemic pulmonary collateral arteries (SPCAs), represents a complex anatomic and surgical spectrum of congenital heart disease. Currently, there is limited evidence on homograft durability after complete correction, which potentially could be affected by anatomic differences in pulmonary vasculature.

**Methods:**

This retrospective single-center study included all 69 consecutive PA-VSD patients (46 with SPCAs, 23 without SPCAs) operated on between 1978 and 2018. The primary interest was in homograft durability after complete repair. Longitudinal echocardiographic homograft function and right ventricular systolic pressure were analyzed with linear mixed-effects models.

**Results:**

The median duration of follow-up was 20 years. Of the 46 patients with SPCAs, 37 (80.4%) underwent biventricular correction at a median age of 2.7 years (interquartile range [IQR], 1.8-6.3 years). Two patients are currently awaiting unifocalization and correction. All 23 patients without SPCAs underwent successful complete correction at a median age of 1.6 years (IQR, 1.1-3.6 years). Freedom from any reintervention after 20 years was 15%. When a homograft was used during correction, freedom from homograft replacement after 20 years was comparable in the 2 groups (*P* = .925), at 32 ± 11% in the SPCA group and 32 ± 13% in the non-SPCA group. Indications for homograft replacement were isolated stenosis (n = 7; 46.7%), isolated regurgitation (n = 3; 20.0%), and mixed stenosis and regurgitation (n = 5; 33.3%) in the SPCA group and isolated stenosis (n = 8; 88.9%) and stenosis and regurgitation (n = 1; 11.1%) in the non-SPCA group. Peak homograft gradient was significantly (*P* = .0003) higher in patients without SPCA, with a comparable rate of progression in the 2 groups. However, the prevalence of severe pulmonary regurgitation (PR) was higher in patients with SPCAs, estimated at 35% at 10 years, compared with 15% in patients without SPCAs.

**Conclusions:**

Homografts used for right ventricular outflow tract reconstruction in patients with PA-VSD, either with or without SPCAs, have similar limited durability. Repeated reintervention is common, and careful follow-up with attention to severe PR is warranted.

## Graphical abstract

**Figure:**
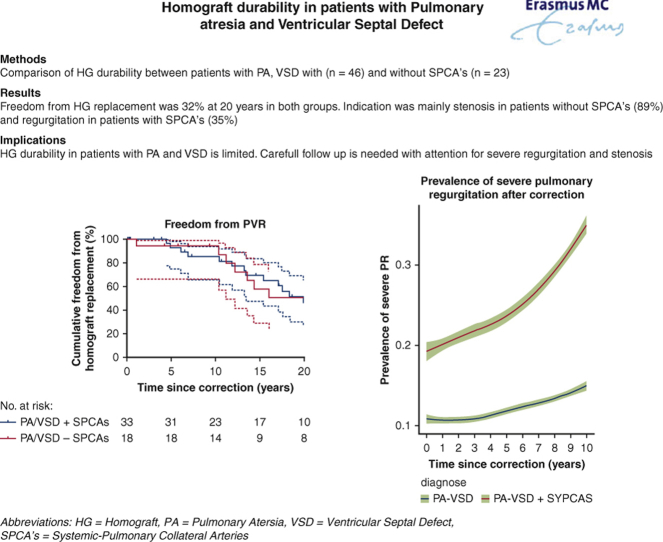

**Figure:**
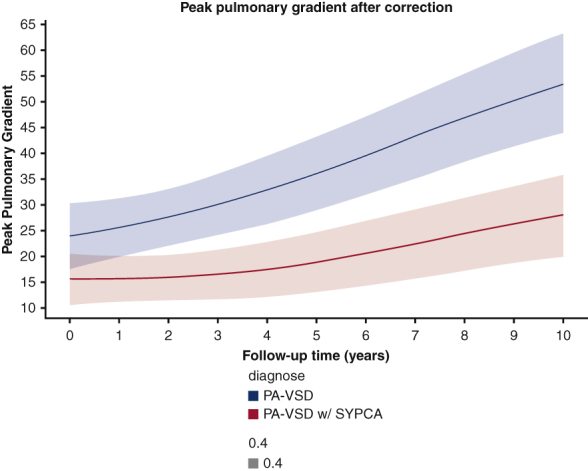
Peak pulmonary gradient in homograft recipients.

**Central Message**

Homografts used for right ventricular outflow tract reconstruction in patients with pulmonary atresia and ventricular septal defect have limited durability. Reintervention is common, and follow-up with attention to pulmonary regurgitation is warranted.

**Perspective**

Our study with a long follow-up on homograft durability in patients with correction of pulmonary atresia and ventricular septal defect with or without systemic-to-pulmonary collateral arteries (SPCAs) demonstrates that repeated reintervention is common. Longitudinal echocardiographic follow-up showed both stenosis (more in the non-SPCA group) and regurgitation (more in the SPCA group). Careful follow-up is therefore warranted.

See Commentary on page 556.

Pulmonary atresia and ventricular septal defect (PA-VSD) with or without systemic pulmonary collateral arteries (SPCAs) are complex congenital cardiac defects. Without surgical intervention, only 75% of patients survive the first year of life.^1^

Varying sources of blood supply are present. In PA-VSD, the pulmonary arterial system is essentially normal but initially duct-dependent, and most often a first step in surgical treatment consists of creating an aortopulmonary shunt.^2^, ^3^ In PA-VSD with SPCAs, the pulmonary arterial system varies in each SPCA-supplied bronchopulmonary segment combined with a varying presence of native pulmonary arteries (NPAs), and the choice of a single-stage or staged approach is an issue.

In both entities, surgical approach and timing are important considerations. In PA-VSD without SPCAs, the approach is aimed at preservation and inclusion of the complete NPAs. This is also the aim in PA-VSD with SPCAs, but unifocalization and rehabilitation of the pulmonary arterial system are necessary.^4^, ^5^ Regardless of the surgical approach, right ventricular outflow tract (RVOT) reconstruction with a valved conduit, often a homograft, is an essential part of biventricular correction. Because patients with incorporated SPCA segments may be expected to have a less compliant pulmonary arterial bed with higher pulmonary artery pressures owing to suture lines and increased pulmonary vascular resistance, homograft durability might be hampered. Homograft durability may be limited, manifesting as severe stenosis, regurgitation, or a combination of both and is associated with reintervention, morbidity, and mortality.^6^^,^^7^ Consequently, in the present study, we aimed to determine homograft durability over our 26-year experience with staged surgical repair of PA-VSD with and without SPCAs.

## Methods

All consecutive patients with PA-VSD with or without SPCAs who underwent correction between 1978 and 2018 at Erasmus University Medical Center were included. Hospital records were reviewed retrospectively after the Medical Ethics Commission reviewed and approved this study (MEC 12-477, approved 7/1/2012). The requirement for individual informed consent was waived.

### Surgical Technique

All patients were discussed in multidisciplinary meetings involving congenital cardiologists, cardiac surgeons, and radiologists. Eligibility for biventricular complete repair was based on anatomic feasibility and invasive evaluation. In most cases, intracardiac anatomy was determined based on a combination of echocardiography, angiography, and computed tomography scans. The Nakata index was calculated for most patients before and after correction.^8^ The right ventricular systolic pressure was obtained through cardiac catheterization or calculated using the simplified Bernoulli equation based on the systolic tricuspid regurgitation jet. A Nakata index of at least 150 was considered favorable for a positive outcome after complete repair but was not used as an exclusion criterion.

Complete single-stage repair with concomitant unifocalization without the use of homografts was performed in a minority of patients (n = 4; 8.5%) operated on between 1978 and 1992. Since then (1992-2018 and the present day), surgical policy has been a staged approach with RVOT reconstruction using a homograft conduit during final repair as early as clinically feasible. Through separate lateral thoracotomies, unifocalization is intended, with intrapulmonary anastomosis if possible to avoid inclusion of proximal segments of SPCAs in the reconstructed pulmonary vascular bed. In our cohort, these procedures were most often (n = 28) completed with an ipsilateral modified Blalock–Taussig shunt (BTS). In cases of a ductal-dependent PA-VSD without SPCAs, neonatal palliation with a modified BTS was applied in all patients, followed later by complete repair.

Correction involved patch closure of the VSD baffling the left ventricle to the aorta and reconstruction of the RVOT with a cryopreserved homograft. No intraoperative flow studies were carried out, and no VSD was deliberately left open. In all cases, intraoperative echocardiography was used to check the adequacy of repair. Primary outcomes were homograft reintervention and replacement.

### Statistical Analysis

Categorical variables are presented as frequency with percentage, and continuous variables are presented as mean with standard deviation or median with range, as appropriate. Time-dependent outcomes are reported using a life tables method for defined periods. Kaplan–Meier plots were created to visualize the occurrence of time-dependent outcomes, and between-group comparisons were made using the log-rank test or Tarone–Ware test as appropriate. Serial echocardiographic measurements of the peak transvalvular homograft gradient and regurgitation grade were analyzed using linear mixed-effects models (Table E1). A significance level of 0.05 was used. Statistical analyses were carried out with SPSS version 24.0 (IBM, Armonk, NY.) and R (R Foundation for Statistical Computing, Vienna, Austria).

## Results

### Interstage Results

**Figure 1:**
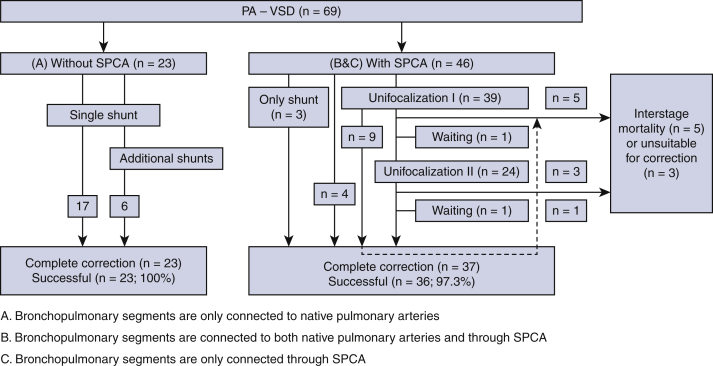
Flow chart of the surgical process for all patients. A, Bronchopulmonary segments are connected only to native pulmonary arteries (NPAs). B, Bronchopulmonary segments are connected both through NPAs and through systemic-to-pulmonary collateral arteries (*SPCAs*). C, Bronchopulmonary segments are connected only through SPCAs. *PA-VSD*, Pulmonary atresia and ventricular septal defect.

Sixty-nine consecutive patients with SPCAs (n = 46) and without SPCAs (n = 23) were been referred to our clinic (Table 1). Among the 39 patients who underwent unifocalization, 29 (74.4%) underwent successful correction at a median age of 2.6 years (interquartile range [IQR], 1.8-5.8 years) (Figure 1). Two patients are currently alive and well and awaiting second unifocalization and complete correction, respectively. All 23 patients without SPCAs achieved correction at a median age of 1.6 years (IQR, 1.1-3.6 years). Of the 46 patients with SPCAs referred to our clinic, 37 underwent attempted correction, which was successful in 36 (97.3%). Therefore, a total of 60 patients underwent complete repair at a median age of 2.2 years (IQR, 1.3-6.1 years), and surgery was successful in 59 (98.3%) (Table 2). In 54 patients, repair involved the use of a homograft.

**Table 1:** Baseline patient characteristics

Characteristic	PA-VSD with SPCAs (N = 46)	PA-VSD without SPCAs (N = 23)
Male sex, n (%)	22 (47.8)	12 (52.2)
Genetic abnormalities, n (%)	12 (26.1)	2 (8.7)
22q11 deletion	12 (26.1)	0 (0)
16p11.2 deletion	0 (0)	1 (4.3)
5q22 duplication	0 (0)	1 (4.3)
Age at first palliation, y, median (range)	0.58 (0.04-21.06)	0.04 (0.01-1.11)
Number of palliative shunts, n (%)		
0	5 (10.9)	0 (0)
1	21 (45.7)	17 (73.9)
2	20 (43.5)	6 (26.1)
Type of palliative shunt, n (%)	61 (100)	29 (100)
Blalock–Taussig shunt	47 (77.0)	22 (75.9)
Waterston shunt	1 (1.6)	5 (17.2)
Potts shunt	1 (1.6)	0 (0)
Central shunt	12 (19.7)	2 (6.9)
Unifocalization, n (%)		NA
Unifocalization	39 (84.8)	
Single stage complete correction	4 (8.7)	
Only shunting	3 (6.5)	
Interstage mortality, n (%)	5 (10.6)	0 (0)
Unsuitable for biventricular repair, n (%)	3 (6.5)	0 (0)
Interstage waiting, n (%)	2 (4.3)	0 (0)
Intention to perform complete correction, n (%)	37 (80.4)	23 (100)

*PA-VSD*, Pulmonary atresia with ventricular septal defect; *SPCAs*, systemic pulmonary collateral arteries.

**Table 2:** Baseline characteristics of patients with complete repair

Characteristic	PA-VSD with SPCAs (N = 37)	PA-VSD without SPCAs (N = 23)
Age, y, mean ± SD; median (range)	5.3 ± 2.7; 2.7 (0.08-30.5)	2.8 ± 2.8; 1.6 (0.4-9.5)
Length, cm, mean ± SD	97 ± 27	81 ± 13
Weight, kg, mean ± SD	15.5 ± 11.4	10 ± 3.4
Creatinine, μmol/L, mean ± SD	34 ± 15	26 ± 6.2
Hemoglobin, mmol/L, median (range)	10 (8-13)	10 (8-12)
Hematocrit, mean ± SD	0.38 ± 0.47	0.39 ± 0.50
CPB time, min, mean ± SD	186 ± 70	171 ± 46
Cross-clamp time, min, mean ± SD	106 ± 35	111 ± 21
Successful surgery, n (%)	36 (97.3)	23 (100)
VSD closure, n (%)	36 (97.3)	23 (100)
Valved conduit RVOT reconstruction, n (%)		
Pulmonary homograft	31 (86.1)	16 (69.6)
Aortic homograft	4 (11.1)	3 (13.0)
Transannular patch	0 (0)	4 (17.4)
Other	1 (2.8)	0 (0)
Homograft diameter (mm), median (range)∗	20 (12-25)	17 (11-24)
Postoperative complications, n (%)		
Death	2 (5.6)	0 (0)
Redo thoracotomy	6 (16.7)	1 (4.3)
For bleeding	4 (11.1)	0 (0)
For other reasons	2 (5.6)	1 (4.3)
Other	4 (11.1)	2 (8.7)

*PA-VSD*, Pulmonary atresia with ventricular septal defect; *SPCAs*, systemic pulmonary collateral arteries; *SD*, standard deviation; *CPB*, cardiopulmonary bypass; *RVOT*, right ventricular outflow tract.

∗ If the homograft was bicuspidalized, the final diameter is reported.

### Outcome

**Figure 2:**
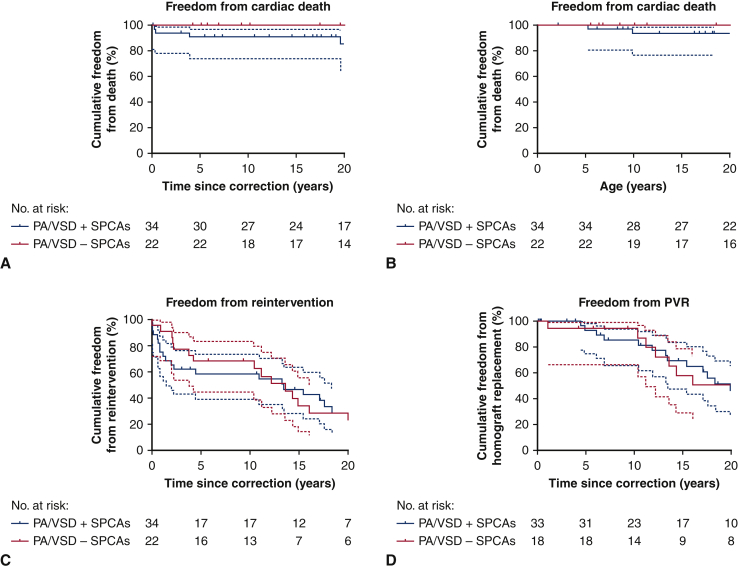
A, Kaplan-Meier plot of freedom from death as a function of time since correction, according to diagnosis. B, Kaplan-Meier plot of freedom from death as a function of age, according to diagnosis. C, Kaplan-Meier plot of freedom from first reintervention after complete correction. D, Kaplan-Meier plot of freedom from pulmonary valve replacement in patients who received an allograft at complete correction. *PA/VSD*, Pulmonary atresia with ventricular septal defect; *SPCAs*, systemic pulmonary collateral arteries; *PVR*, pulmonary valve replacement.

Two patients (5.6%) from the SPCA group died in the hospital shortly after surgical correction. Follow-up was complete for 56 hospital survivors (98.2%) after correction, after a median period of 20.6 years (range, 0.12-38.3 years) and all within 2 years of study closure. One patient was lost to follow-up immediately after correction owing to emigration to her country of origin. There were 5 late deaths (8.9%) after a median 3.9 years (range, 0.3-26.1 years) since correction, at a median age of 25.8 years (range 5-43 years). All 5 late deaths occurred in the SPCA group, including 4 cases of sudden unexplained and unexpected death at 3 months, 5 months, 4 years, and 20 years after correction. The fifth patient died at 26 years after correction from pneumosepsis with right heart failure. Thus, survival at 20 years after correction was 100% in patients without SPCAs and 86 ± 6% in patients with SPCAs (Figure 2).

### Hemodynamic Outcomes

**Figure 3:**
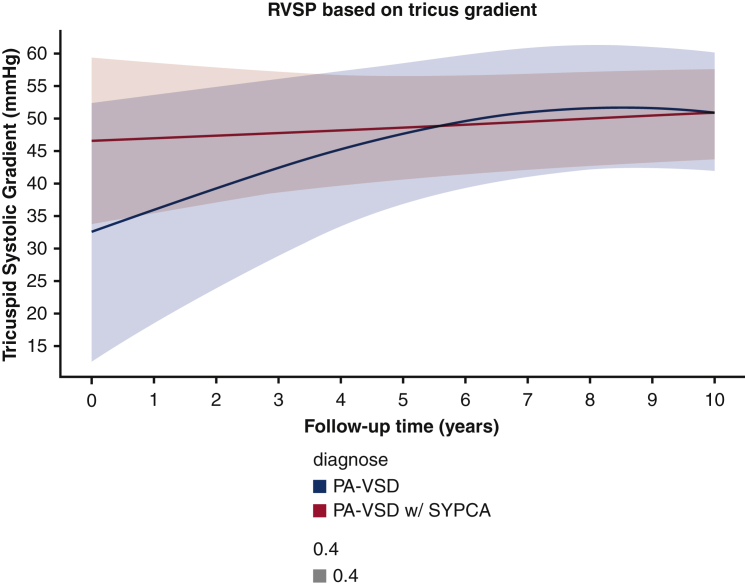
Peak right ventricular systolic pressure (*RVSP*) after correction. *PA-VSD*, Pulmonary atresia with ventricular septal defect; *SYPCA*, systemic pulmonary collateral arteries.

**Figure 4:**
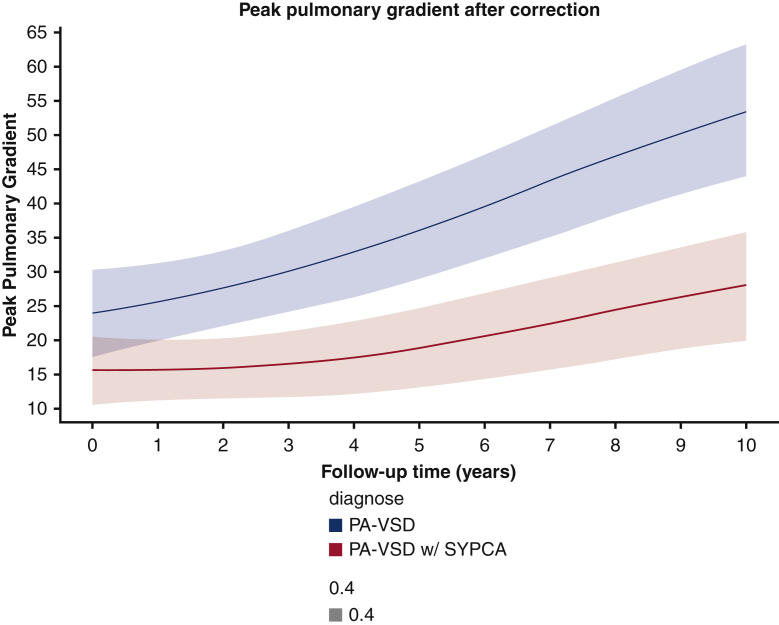
Peak pulmonary gradient in homograft recipients. *PA-VSD*, Pulmonary atresia with ventricular septal defect; *SYPCA*, systemic pulmonary collateral arteries.

**Figure 5:**
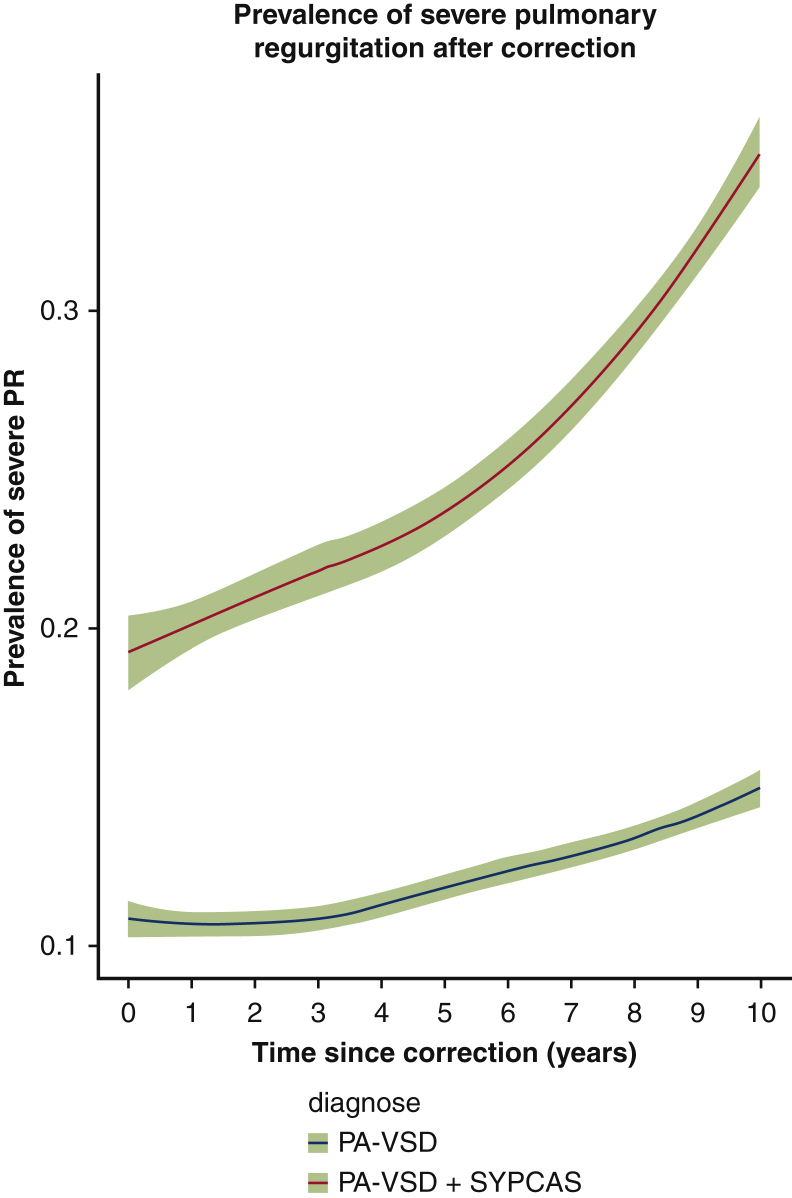
Prevalence of severe pulmonary regurgitation (*PR*) in homograft recipients. *PA-VSD*, Pulmonary atresia with ventricular septal defect; *SYPCAS*, systemic pulmonary collateral arteries.

Right ventricular systolic pressure was similar in the 2 groups (*P* = .247) (Figure 3). The number of patients at risk and measurements available at each year are reported in Table E2. The peak homograft gradient was significantly higher (*P* = .0003) in patients without SPCA, with a comparable rate of progression in the 2 groups (Figure 4); however, the prevalence of severe pulmonary regurgitation (PR) at 10 years was higher in the patients with SPCAs, estimated at 35% at 10 years, versus 15% in those without SPCAs (Figure 5).

Available hemodynamic invasive data in the patients without SPCAs showed low pulmonary artery pressure before correction. In 16 patients, at longer-term (median time since correction, 12 years; range, 2-23 years), the median invasive systolic pulmonary artery pressure was 28 mm Hg (range, 14-64 mm Hg), and the median pressure gradient across the homograft was 46 mm Hg (range 8-66 mm Hg). Only 2 patients had pulmonary artery branch stenosis with low pressure distally.

Pulmonary artery pressures were significantly higher in the patients with SPCAs. In 18 patients with a median time after correction of 6 years (range, 0-26 years), the median systolic pulmonary artery pressure was 50 mm Hg (range, 23-95 mm Hg), and the median pressure gradient across the homograft was only 12 mm Hg (range, 0-56 mm Hg).

### Late Events: Reintervention

There were 63 reinterventions on the RVOT in 38 patients (67.9%), including 37 surgical interventions and 26 transcatheter interventions after correction (Table 3). Freedom from any intervention in patients with SPCAs was 47 ± 9% after 10 years and 23 ± 9% after 20 years. In patients without SPCA, these values were 34 ± 11% and 9 ± 7%, respectively. In patients in whom a homograft was inserted as part of correction, freedom from homograft replacement after 20 years was comparable in the 2 groups (32 ± 11% in the SPCA group and 32 ± 13% in the no-SPCA group; Tarone–Ware test, χ^2^ = 0.009; *P* = .925). In the SPCA group, indications for homograft replacement were isolated stenosis in 7 patients (46.7%), isolated regurgitation in 3 (20.0%), and mixed stenosis and regurgitation in 5 (33.3%). In the patients without SPCA, isolated stenosis was the indication in 8 patients (88.9%), and 1 patient (11.1%) had a mixed hemodynamic profile.

**Table 3:** Reintervention in hospital survivors after correction

	Number of events/LOR	
	PA-VSD with SPCAs (N = 34)∗	PA-VSD without SPCAs (N = 22)
Reinterventions†		
Reintervention per patient, median (range)	1 (0-8)	1 (0-3)
Patients with multiple reinterventions, n	10	7
Surgical procedures, n (%)‡	22 (3.8)	15 (3.4)
PVR	14 (2.4)	12 (2.7)
Plasty confluens/branch PA	6 (1.0)	8 (1.8)
Residual VSD closure	6 (1.0)	4 (0.9)
Transcatheter procedures, n (%)‡	17 (2.9)	9 (2.0)
Balloon angioplasty	15 (2.6)	9 (2.0)
With stenting	13 (2.2)	7 (1.6)
Transcatheter PVR	3 (0.5)	2 (0.5)
Pacemaker or ICD placement	0 (0)	0 (0)
Reinterventions on, n (%)†		
RVOT/MPA, including valve	20 (2.4)	18 (4.1)
Branch PA	19 (3.3)	14 (3.2)
Residual SPCA	0 (0)	NA
Residual VSD	6 (1.0)	4 (0.9)
Events, n (%)		0 (0)
Late death	5 (0.9)	0 (0)
Noncardiac death	1 (0.2)	0 (0)
Cardiac death	4 (0.7)	0 (0)
Valve-related	0 (0)	0 (0)
SUUD	4 (0.7)	0 (0)
Stroke	0 (0)	0 (0)
TIA	0 (0)	0 (0)
Bleeding	0 (0)	0 (0)
Endocarditis	2 (0.3)	3 (0.7)

*LOR*, Linearized annual occurrence rate since correction; *PA-VSD*, pulmonary atresia with ventricular septal defect; *SPCAs*, systemic pulmonary collateral arteries; *PVR*, pulmonary valve replacement; *PA*, pulmonary artery; *VSD*, ventricular septal defect; *ICD*, implantable cardioverter defibrillator; *RVOT*, right ventricular outflow tract; *MPA*, main pulmonary artery; *SUUD*, sudden unexplained unexpected death; *TIA*, transient ischemic attack.

∗ Correction was attempted in 37 patients; 1 was unsuccessful, and 2 patients died.

† Reinterventions on different anatomic structures during the same session are reported separately.

‡ Concomitant procedures are reported separately.

All patients who underwent correction without a homograft (ie, a Hancock prosthesis in 2 and transannular patch in 3), received a homograft at a later stage. Surgical reintervention due to restenosis of branch pulmonary arteries was performed in 11 patients (19.6%). Closure of a residual VSD was performed in 10 patients (17.9%). One left-sided modified BTS was reinserted 9 months after correction in a 3-year-old boy owing to restenosis and hypoplasia of the left pulmonary artery with virtually no flow to the left lung. Of the 4 early patients who underwent single-stage unifocalization and correction, 1 patient is still free from reintervention and in good clinical condition 27 years after correction. The other 3 patients underwent reinterventions including pulmonary valve replacement (PVR). One patient who underwent correction at age 6 years died suddenly at age 26 years due to presumed rhythm disturbances. Another female patient who underwent single-stage correction at age 9 years with a Hancock prosthesis underwent PVR with an aortic homograft 11 years later. Seventeen years later, she underwent a Bentall procedure due to severe aortic regurgitation and RVOT reconstruction with a mechanical prosthesis due to severe stenosis and regurgitation of the homograft. She is currently in good clinical condition. The fourth patient underwent complete correction at age 1 month with an aortic homograft, which was replaced 5 years later with concomitant reconstruction of the confluent pulmonary arteries.

## Discussion

Homografts used for RVOT reconstruction in patients with PA-VSD, both with and without SPCAs, have limited durability. Durability is limited primarily by progressive regurgitation in patients with PA-VSD and SPCAs and by progressive stenosis in those with PA-VSD without SPCAs.

### Homograft Durability

The main shortcoming of homografts is durability, which appears to be especially limited in young patients.^9^, ^10^, ^11^ It is reasonable to expect most homografts to eventually fail due to severe stenosis, regurgitation, or both. Reports on long-term outcomes after PA-VSD correction emphasizing homograft durability are sparse, but similar high reintervention rates have been reported.^12^^,^^13^ In our series, the majority of patients in the SPCA group underwent at least 1 reintervention, and almost one-half of them underwent multiple reinterventions. In both groups, PVR was the most frequently performed surgical reintervention. Increased right ventricular (RV) afterload, which is occasionally present in patients with unifocalized SPCAs, can further limit durability by exerting additional tissue stress. Mainwaring and colleagues^13^ reported a negative correlation between pulmonary artery pressure as directly measured by a catheter postoperatively and aortic homograft durability. Our present results show that both RV systolic pressure and the homograft replacement rate were comparable after correction in our patients with SPCAs and those without SPCAs. Although the peak gradient was significantly higher in the patients without SPCAs, a larger proportion of PVR was indicated by severe PR or mixed stenosis and regurgitation in the patients with SPCAs, which equalized the total PVR rates between the groups. This is remarkably similar to the results presented by Mainwaring and colleagues, in which significant PR was present in the vast majority of failing homografts.^13^ In a swine model, Petit and colleagues^14^ found that a reduction in pulmonary artery pressure can lead to a reduced regurgitation fraction.

The exact nature of the development of high RV systolic pressure over time since correction remains both a question and problem. Our results based on a staged approach indicate stable RV systolic pressure for at least 10 years after correction, while taking into account that 32% of patients underwent at least 1 catheter intervention on the pulmonary vasculature since correction. Long-term repeated analyses of RV systolic pressure are scarce, but those available generally report stable pressures.^15^ Nonetheless, the extent to which the unifocalized pulmonary vasculature and RV systolic pressure in humans contributes to an increased prevalence of severe PR and homograft durability remains uncertain. Our hemodynamic data support the difference in pulmonary artery pressure between the 2 groups. It can be speculated that the higher pressures in patients with SPCAs lead to more regurgitation, and that the homograft degeneration in patients without SPCAs leads to more stenosis in this group.

### Surgical Policy

At both ends of the classification spectrum proposed by Castaneda and colleagues^16^ and the Society of Thoracic Surgeons Congenital Heart Surgery Nomenclature and Database Project,^17^ surgery is relatively straightforward. The surgical approach of patients with pulmonary segments exclusively connected to NPAs is complete correction, sometimes in a single procedure with good short- and long-term results similar to our current findings.^15^^,^^18^ Similarly, if systemic collaterals are the sole source of pulmonary blood flow, unifocalization is an essential part of correction. Carrillo and associates^19^ reported acceptable reintervention rates in 28 patients without NPAs who underwent single-stage complete correction. Follow-up was modest, however, and long-term reliability on these SPCAs remains questionable.^19^ The durability of SPCAs in patients with moderately matured NPAs, which probably represent the majority of the population, is unclear. The value of unifocalization in this population has been questioned based on reportedly limited growth capacity and unpredictable durability of SPCAs.^2^^,^^20^, ^21^, ^22^ Our surgical policy is based on staged unifocalization, differing from others^5^^,^^19^^,^^23^, ^24^, ^25^ in that we do not pursue single-stage unifocalization and correction at the earliest onset, even in cases of complete dual supply of pulmonary blood flow. Our strategy includes combined elements of staged unilateral unifocalization with an ipsilateral or central shunt before complete correction, comparable to the policy of the Cleveland Clinic.^26^ However, this approach results in more suture lines in the pulmonary arterial bed and possibly longer exposure of pulmonary arterial segments with increased pulmonary arterial pressure, which may hamper the longevity of homografts used in correction. This effect was not observed in our study, however.

### Study Strengths and Limitations

We have presented the long-term durability of homografts in patients with pulmonary atresia after a uniform strategy of correction with extensive centralized follow-up. Advanced statistical techniques were used to analyze repeatedly assessed echocardiographic function of homografts and RV systolic pressure. Limitations are inherent to the study's retrospective and single-center nature with a modest patient number, leading to some missing data and the potential for bias.

## Conclusions

**Figure 6:**
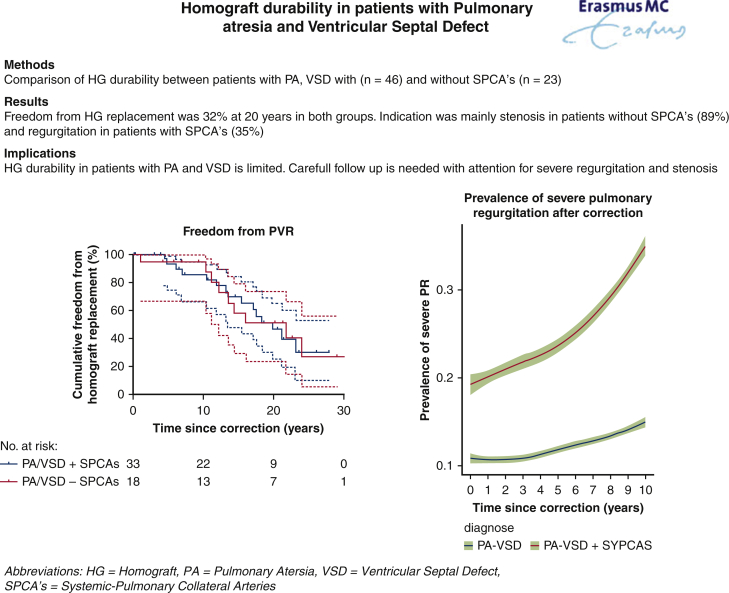
Comparison of homograft (*HG*) durability between patients with pulmonary atresia with ventricular septal defect (*PA-VSD*) with systemic pulmonary collateral arteries (*SPCAs*) (n = 46) and without SPCAs (n = 23). Long-term results with freedom from HG replacement of 32% at 20 years in both groups but with different indications show more stenosis in the group without SPCAs and more regurgitation in the group with SPCAs.

Homograft durability in patients with PA-VSD with or without SPCAs is comparable; however, significant PR is more prevalent in patients with unifocalized SPCAs. A multistage approach with staged unifocalization and concomitant shunting can lead to satisfactory repair rates in patients with PA-VSD and SPCAs. Figure 6 is a graphical abstract with this conclusion.

### Conflict of Interest Statement

The authors reported no conflicts of interest.

The *Journal* policy requires editors and reviewers to disclose conflicts of interest and to decline handling or reviewing manuscripts for which they may have a conflict of interest. The editors and reviewers of this article have no conflicts of interest.

## Figures and Tables

**Table E1:** Linear mixed model design

Effects	Factors
Fixed effects	Diagnose (ie, with or without SPCAs) Sex (ie, male or female) Age at correction (y) Type of homograft (aortic or pulmonary) Weight at correction (kg) Length at correction (cm)
Random effects	Time since operation in years Time since operation in years^2^ (second-order derivative) Time since operation in years^3^ (third-order derivative)

*SPCAs*, Systemic pulmonary collateral arteries

**Table E2:** Number of patients at risk and available echocardiography measurements

Time, y	Number of patients at risk	Number of echocardiography measurements available
0	51	655
1	49	608
2	49	548
3	49	511
4	47	456
5	44	411
6	43	370
7	40	338
8	38	300
9	38	273
10	37	245
